# Turtling the Salamander: Tail Movements Mitigate Need for Kinematic Limb Changes during Walking in Tiger Salamanders (*Ambystoma tigrinum)* with Restricted Lateral Movement

**DOI:** 10.1093/iob/obab029

**Published:** 2021-10-25

**Authors:** Christine M Vega, Miriam A Ashley-Ross

**Affiliations:** Department of Biology, Wake Forest University, 1834 Wake Forest Road, Winston-Salem, NC 27109, USA; Department of Biology, Wake Forest University, 1834 Wake Forest Road, Winston-Salem, NC 27109, USA

## Abstract

Lateral undulation and trunk flexibility offer performance benefits to maneuverability, stability, and stride length (via speed and distance traveled). These benefits make them key characteristics of the locomotion of tetrapods with sprawling posture, with the exception of turtles. Despite their bony carapace preventing lateral undulations, turtles are able to improve their locomotor performance by increasing stride length via greater limb protraction. The goal of this study was to quantify the effect of reduced lateral flexibility in a generalized sprawling tetrapod, the tiger salamander (*Ambystoma tigrinum*). We had two potential predictions: (1) either salamanders completely compensate by changing their limb kinematics, or (2) their performance (i.e., speed) will suffer due to the reduced lateral flexibility. This reduction was performed by artificially limiting trunk flexibility by attaching a 2-piece shell around the body between the pectoral and pelvic girdles. Adult tiger salamanders (*n* = 3; SVL = 9–14.5 cm) walked on a 1-m trackway under three different conditions: unrestricted, flexible shell (Tygon tubing), and rigid shell (PVC tubing). Trials were filmed in a single, dorsal view, and kinematics of entire midline and specific body regions (head, trunk, tail), as well as the fore and hind limbs, were calculated. Tygon individuals had significantly higher curvature than both PVC and unrestricted individuals for the body, but this trend was primarily driven by changes in tail movements. PVC individuals had significantly lower curvature in the trunk region compared with unrestricted individuals or Tygon; however, there was no difference between unrestricted and Tygon individuals suggesting the shells performed as expected. PVC and Tygon individuals had significantly higher curvature in the tails compared with unrestricted individuals. There were no significant differences for any limb kinematic variables among treatments including average, minimum, and maximum angles. Thus, salamanders respond to decreased lateral movement in their trunk by increasing movements in their tail, without changes in limb kinematics. These results suggest that tail undulations may be a more critical component to sprawling-postured tetrapod locomotion than previously recognized.

## Introduction

Once early tetrapods began making forays onto land, significant changes to the musculoskeletal system were necessary to support their body weight out of the water and their enhanced terrestrial locomotion. As tetrapods became more terrestrial, the axial skeleton became more robust and different regions along the length of the vertebral column became specialized for different functional roles (Long and Gordon 2004). These anatomical changes to the axial skeleton reflect the different mechanical demands experienced by parts of the vertebral column as well as by different taxa (Kardong 2012). For example, one significant functional change that resulted from tetrapods becoming more terrestrial is the transfer of propulsive forces through the limbs and limb girdles to the vertebral column (Kardong 2012).

Salamanders are commonly viewed as a model for studies of the terrestrial locomotion of early tetrapods because their sprawling limb posture and generalized body plan are thought to resemble those of many of the earliest vertebrates to move over land (Edwards 1989; Ashley-Ross and Bechtel 2004). During swimming, salamanders propel themselves through undulations of the trunk and tail, passing a traveling wave down the body axis; in contrast, walking salamanders rely heavily on their limbs for propulsion and generate a standing wave with their body axis (Frolich and Biewener 1992).

Epaxial muscles are active to produce these motions during both swimming and walking but display distinct activation patterns in each behavior. During swimming, activation of epaxial muscles on one side of the trunk allows the body to create a concave curve contributing to the generation of a traveling wave (Frolich and Biewener 1992; Carrier 1993). However, during walking, hypaxial muscles on both sides of the trunk are active at different points with the obliquus externus superficialis and profundus muscles active on the bending side, and the obliquus internus and transversalis muscles active on the side opposite the bending (Carrier 1993; Bennett et al. 2001). These distinct muscle activation patterns have functional consequences for the roles of hypaxial muscles in swimming and walking: the hypaxial muscles contribute to lateral bending while swimming, but are used for stabilizing the trunk against long-axis torsion during terrestrial walking (Carrier 1993; Bennett et al. 2001). The lateral undulations generated by the activation of the hypaxial muscles also increase stride length and may be used to modify the stiffness of the trunk and provide a mechanism for adjusting gaits and stabilizing against gravitational and muscular forces (Deban and Schilling 2009; Knuesel and Ijspeert 2011; Wang et al. 2018).

Kinematic modifications can enhance certain aspects of performance in dynamic environments giving animals fine-tuned control over their locomotion. In tetrapods, flexibility of the spine is thought to be important for speed adjustments, stabilization, turning trajectories, and energy efficiency (Wang et al. 2018). For example, mammalian tails are known to be important for body stabilization and balance during locomotion for cats (Walker et al. 1998), dogs (Wada et al. 1993), and many rodent species (Bartholomew and Caswell 1951). By contrast, the significance of tail movements for the locomotion of sprawling tetrapods is less clear in the literature. The tail can serve as a fifth limb that increases the odds of slip recovery during climbing (Jusufi et al. 2008).

In sprawling tetrapods, the caudofemoralis muscle is relatively large and passes from the femur to the tail and serves to retract the femur to generate propulsion (Gatesy 1990). When the hindfoot is placed on the ground, the tail is typically deflected toward the same side. As stance phase progresses, the tail is pulled over to the opposite side that also has the effect of stretching the caudofemoralis that could improve hind limb muscle function by increasing propulsive forces generated during locomotion (Irschick and Jayne 1999). The caudofemoralis retracts the hind limb during stance phase (Reilly 1994; Irschick and Jayne 1999). The muscular tails of crocodilians, which provide propulsive power during swimming and aquatic attacks, may compromise locomotion by interfering with hind limb movements (Willey et al. 2004). The substantial weight of the tail shifts the center of mass caudally in alligators (*Alligator mississippiensis*), which affects the functional role of the hind limbs during locomotion. The hind limbs of alligators are involved in body weight support and also have much higher propulsive forces relative to counteracting the braking effect of the dragging tail (Willey et al. 2004).

Turtles, like other sprawling-postured tetrapods, rely on their limbs for generating propulsion for both aquatic and terrestrial locomotion. However, unlike the locomotion of other sprawling tetrapods, the locomotion of turtles is not characterized by significant lateral undulations of the vertebral column because the trunk vertebrae are fused to a bony carapace. Despite what may seem to be a severe morphological limitation, turtles can be found in both terrestrial and aquatic environments and have modified other aspects of kinematics to optimize locomotor performance given the constraints of their shells. For example, forelimb protraction is much higher in turtles relative to other sprawling tetrapods (Walker 1971; Pace et al. 2001; Schmidt et al. 2016). In this context, turtles provide a useful point for comparison for understanding how axial movements contribute to locomotion, because they allow observations of locomotion under conditions in which such motions are limited. However, because turtles possess numerous additional specializations, it can be difficult to tease apart whether features of turtles’ locomotion are specifically due to restriction of axial movements. Instead, insight might be gained by restricting the trunk movements of a generalized tetrapod, so that locomotion could be compared under restricted and unrestricted conditions, without other confounding factors. Previous studies have also implemented this perspective of modifying a standard taxon to isolate the role of a particular feature during locomotion (Jagnandan and Higham 2017; Mayerl et al. 2019b).

The goal of this study was to quantify the effect of reduced lateral flexibility on locomotion in a sprawling tetrapod. We addressed this goal by reducing the flexibility of the vertebral column using tiger salamanders (*Ambystoma tigrinum*) as a model. Salamanders were filmed walking under the following conditions: unrestricted, with a flexible tube (Tygon) surrounding the trunk, and with a rigid tube surrounding the trunk (polyvinyl chloride (PVC)). We had two potential predictions: (1) salamanders can completely compensate (maintain similar speed performance) by changing their limb kinematics, or (2) their performance (speed) will suffer. We also predicted that mean absolute trunk curvatures should decrease with the rigid shell, and there should be no difference between unrestricted salamanders and those with a flexible shell.

## Methods

### Experimental animals

Tiger salamanders (snout-vent length (SVL) = 9–14.5 cm) were purchased from a commercial vendor (Charles D. Sullivan Inc., Nashville, TN). All experimental trials were performed with three metamorphosed, adult individuals. Salamanders were housed individually in aquaria with access to a terrestrial landing and kept on a 12:12 h light:dark cycle. All procedures were approved by the Wake Forest University IACUC (A13-203 and A16-171).

### Description of experimental conditions and data collection

Tiger salamanders (*n* = 3) walked on a 1-m trackway under three different “shell” conditions: unrestricted (UR), Tygon shell (TYS), and PVC shell (PVCS). While PVC tubing was completely rigid and did not allow any bending, Tygon tubing (often used as standard clear lab or tank tubing) can be made of a variety of base materials, but bends easily and resists compression. The Tygon and PVC tubing were cut longitudinally into half circles, fit around the mid body such that their presence did not interfere with the mobility of the limbs, and secured with two zip ties. The length of the shells was less than the distance between the limb girdles of the salamanders, so the shells did not impede limb movement. The zip ties were secured around the circumference of the salamander's trunk. The shells were tight enough so that salamanders could not easily slip out of them, but did not cause the skin to catch between the two half circles. The purpose of the shell was to restrict lateral movement of the vertebral column between the girdles. The UR condition served as a no-treatment control to record normal kinematic patterns. The TYS condition served as a positive control to ensure the salamanders’ locomotive performance was not hindered simply by having additional weight around their trunk, given that the flexible Tygon tubing did not restrict lateral movements of the vertebral column. Thus, we expected the Tygon shell treatment to have similar results to the UR treatment.

Trials were filmed at 30 frames per second in a single, dorsal view using a Kodak PlaySport camera (Kodak Company, Rochester, NY). A 1 cm × 1 cm grid was visible on the trackway for scaling purposes. A trial was defined as a continuous walking sequence of at least two strides. Animals selected their own speeds for all trials and the range of speeds for UR, TYS, and PVCS were 0.02–0.13 meter per second (mps), 0.02–0.06 mps, and 0.02–0.06 mps, respectively. Despite the range of speeds observed, salamanders used a walking gait for all filming trials. The order in which treatments were introduced was randomized and salamanders were gently pinched at the base of the tail to entice them to begin walking. Video recordings did not exceed 1 h and salamanders were permitted to rest in between trial attempts to control for fatigue. Salamanders were only used for one treatment per filming day.

### Analysis of curvature and kinematic variables

**Fig. 1:**
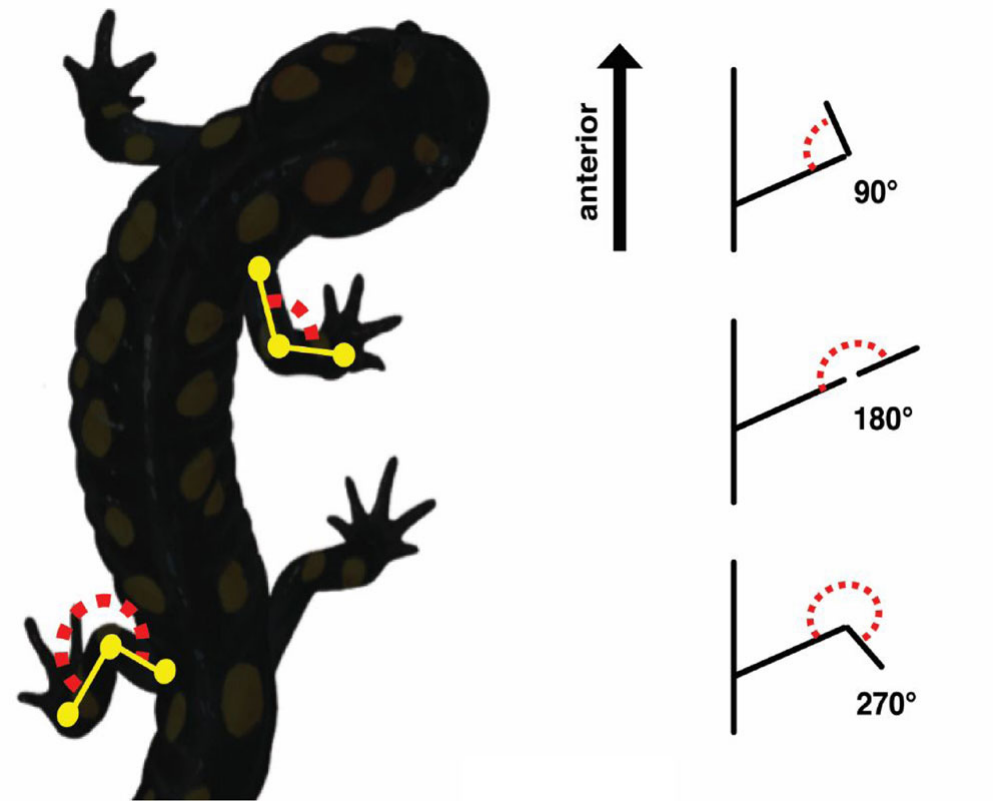
Diagram of elbow and knee angles and interpretation of angle values. Elbow and knee angles for the fore and hind limbs were calculated from two-dimensional coordinate data. An angle less than 180° means the distal portion of the limb is oriented anteriorly and an angle greater than 180° means the distal portion of the limb is oriented posteriorly.

For each individual stride, joints on the fore (wrist, elbow, and shoulder) and hind (ankle, knee, and hip) limbs were digitized frame by frame using DLTdataViewer5 (Hedrick 2008). These two-dimensional coordinate data were used to calculate elbow and knee angles for the limbs for each frame. An angle of 180° means that the limbs are extended straight out from the body. An angle less than 180° means the distal portion of the limb is oriented anteriorly and an angle greater than 180° means the distal portion of the limb is oriented posteriorly (Fig. 1). Mean, minimum, and maximum elbow and knee angles were calculated for all treatments. A LOESS (locally estimated scatterplot smoothing) curve was fit to the angle calculations for each stride individually. We chose LOESS in order to smooth the inherent frame-to-frame error associated with digitizing using a span value of 0.3 and assessing the fit both visually and with a sensitivity analysis. From this curve, we calculated the joint angle and its 95% confidence interval at 0%, 25%, 50%, 75%, and 100% of the stride cycle.

Lateral movement of the vertebral column was quantified from curvature. To calculate curvature, 16 points along the midline of each salamander from the snout to the end of the tail were tracked frame by frame. Mean absolute curvatures were calculated from the shape of the midline along the body length and across the head (including the neck), trunk, and tail regions using a custom MATLAB (MathWorks, Natick, MA) routine that was originally used to assess fin-ray curvature (Chadwell et al. 2012). A previous study determining whether a rigid body limits maneuverability discusses ways to calculate curvature (Walker 2000). Instantaneous curvature (κ), a measure of the rate of rotation of the tangent path, was defined by the parametric function:

$$\begin{equation*}\kappa = \left| {x^{\prime} y^{\prime\prime} - y^{\prime} x^{\prime\prime}} \right|/{\left[ {{{\left( {x^{\prime} } \right)}^2} + {{\left( {y^{\prime} } \right)}^2}} \right]^{3/2}},
\end{equation*}$$

where ′ and ″ denote the first and second derivative of *x* or *y* that relate to the distance along the turning curve; derivatives were estimated using a quintic spline (Walker 2000). Curvatures were calculated for total body length as well as for individual regions to determine whether curvature in other body areas was used to compensate for the restriction in the trunk. Curvature was calculated for the head, trunk, and tail regions by using measurements of the individual salamanders to determine length cutoffs for each body region. Greater curvature values indicate more lateral bending. We used a similar approach to analyze the curvature data as with the kinematic data above. First, we used fifth-order polynomial regression, and omitted any strides with an *R*^2 ^< 0.5. Then, within each treatment group, we averaged the polynomial coefficients from each individual stride. This summary model for each treatment group was used to calculate the mean curvatures and their standard errors at 25%, 50%, and 75% of the stride cycle. Assessing only body curvature can obscure the effects of the shell conditions within body regions; thus, curvature was also analyzed for each body region (head, trunk, and tail) individually.

Summary models for 0% and 100% of the stride were not included for analysis due to the nature of the extreme ends of polynomial functions.

### Statistical analysis

All video trials (*n* = 86) were used in the analysis of both kinematic and midline variables as described above. To compare all locomotor trials, the duration of each cycle was converted to a percentage since not all cycles were of the same absolute duration. All variables were compared over multiple strides from three individuals. All calculations and statistical analyses were performed in R (v. 3.4.3).

Because of the circular/periodic nature of our kinematic data, we used circular statistics to evaluate differences in elbow and knee angles among treatment groups at 0%, 25%, 50%, 75%, and 100% of the stride cycle. A Bayesian approach was used to calculate circular means and to estimate the highest posterior density (HPD) interval. Each individual salamander was included as the random effect to allow for potential inherent differences in individuals. We implemented the analysis using the R package *bpnreg* with 10,000 iterations after 1000 burn-in iterations (Cremers and Klugkist 2018). Differences among treatment groups were deemed significant if their HPDs did not overlap.

We used a general linear mixed model to test for differences in curvature among the treatment groups at 25%, 50%, and 75% of the stride cycle. Individual was included as a random effect, to allow for potential inherent differences among individuals. Post-hoc differences were assessed using Tukey's honestly significant difference (HSD), with Holm–Bonferroni correction for multiple comparisons. Curvature data were log-transformed prior to analysis, and significance was assessed at the ∝ = 0.05 level.

## Results

### Curvature variables

**Fig. 2:**
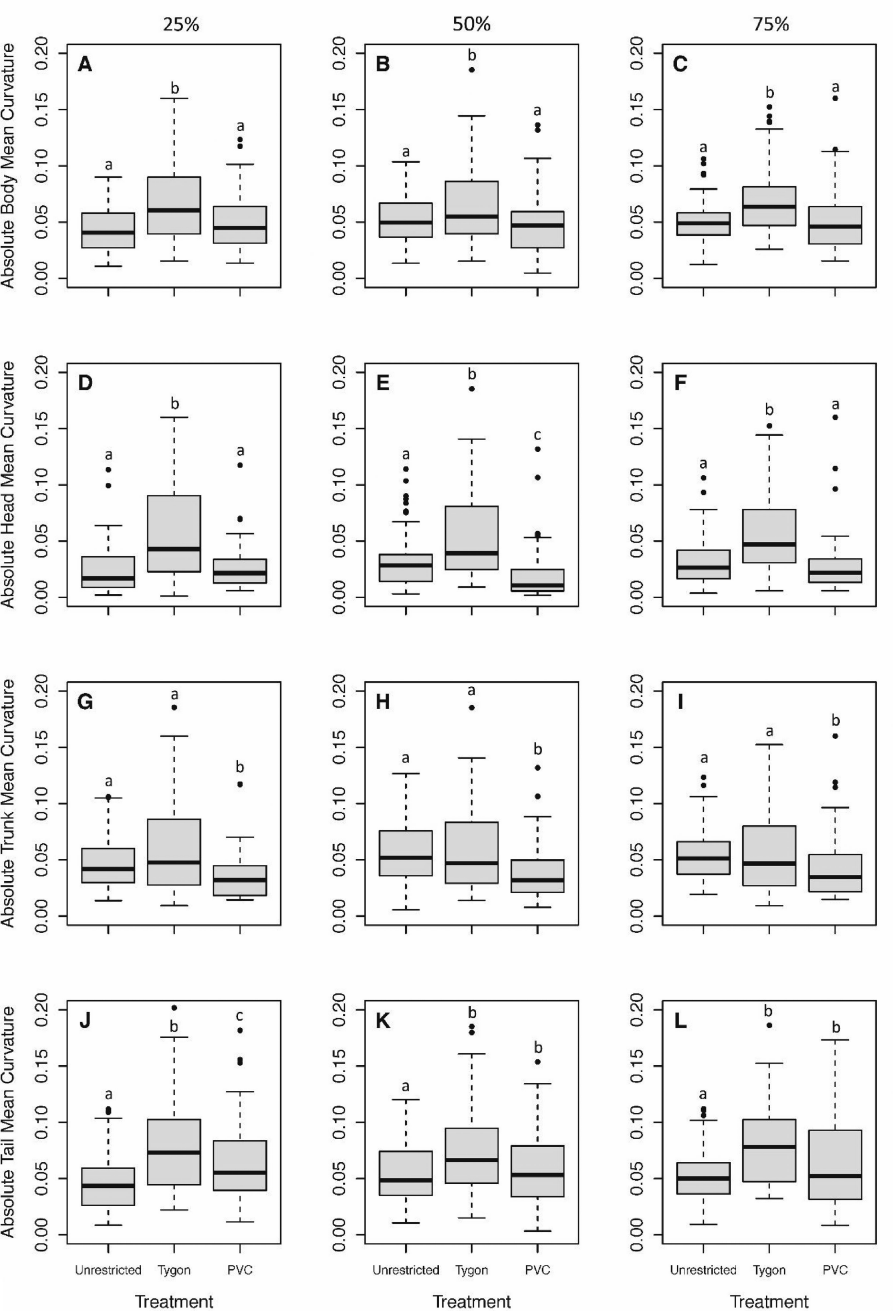
The absolute mean curvature (κ) for Unrestricted, Tygon, and PVC salamanders at 25%, 50%, and 75% of the stride cycle for the body, head, trunk, and tail. At 25% **(A)**, 50% **(B)**, and 75% **(C)** of the stride cycle, TYS individuals had significantly higher curvature than both PVCS and unrestricted individuals for the entire body and there was no difference between UR and PVCS individuals. At 25 **(D)** and 75% **(F)** of the stride cycle, TYS individuals had significantly higher curvature than both PVCS and UR individuals in the head region. At 50% **(E)** of the stride cycle, there were significant differences among all three treatments; TYS individuals had the highest curvature followed by UR and PVCS individuals. At 25% **(G)**, 50% **(H)**, and 75% **(I)** of the stride cycle, PVCS individuals had significantly lower curvature than both TYS and UR individuals in the trunk region. There was no significant difference in curvature between UR and TYS individuals. These results confirm that the PVCS was rigid enough to limit lateral movements in the trunk region and the TYS was flexible enough to permit them. At 25% **(J)**, 50% **(K)**, and 75% **(L)** of the stride cycle, TYS and PVCS individuals both had significantly higher curvature in the tail. At 25% of the stride cycle, TYS individuals also had significantly higher curvature in the tail than PVCS individuals. Salamanders seem to increase lateral movements in their tails when their trunks are restricted.

At 25%, 50%, and 75% of the stride cycle, TYS individuals had significantly higher absolute mean body curvature than both PVCS and UR individuals (Fig. 2A–C; Tables 1, 2 and 3). There was no difference in absolute mean body curvature for those % strides between UR and PVCS individuals (Fig. 2A–C).

**Table 1:** Stride sample size by restriction treatment and individual

	Number of strides by individual		
	C	E	F
Unrestricted	46	53	63
Tygon	40	28	10
PVC	30	56	23

Multiple video trials per salamander were filmed per treatment. The total number of strides analyzed by restriction treatment and individual from these video trials are reported in the table above.

**Table 2:** Mean absolute mean curvature (κ) values and standard error for body regions (body, head, trunk, and tail) and treatments (UR, TYS, and PVCS)

		Body	Head	Trunk	Tail
Unrestricted	25%	0.043 ± 0.001	0.024 ± 0.002	0.046 ± 0.002	0.044 ± 0.002
	50%	0.052 ± 0.002	0.031 ± 0.002	0.055 ± 0.002	0.054 ± 0.002
	75%	0.049 ± 0.001	0.030 ± 0.002	0.053 ± 0.002	0.052 ± 0.002
Tygon	25%	0.069 ± 0.004	0.058 ± 0.006	0.059 ± 0.005	0.080 ± 0.005
	50%	0.066 ± 0.004	0.054 ± 0.006	0.058 ± 0.004	0.076 ± 0.005
	75%	0.069 ± 0.004	0.057 ± 0.006	0.059 ± 0.005	0.081 ± 0.004
PVC	25%	0.051 ± 0.004	0.028 ± 0.004	0.037 ± 0.003	0.066 ± 0.006
	50%	0.005 ± 0.004	0.023 ± 0.005	0.038 ± 0.004	0.064 ± 0.007
	75%	0.053 ± 0.005	0.031 ± 0.005	0.044 ± 0.004	0.066 ± 0.007

Mean values ± standard error of absolute mean curvature for tiger salamanders (*n* = 3 individuals). Absolute mean curvature was calculated along the midline of the salamander for the entire body as well as separately for the head, trunk, and tail regions.

**Table 3:** Results of a general linear mixed effect model performed on absolute mean curvature values for Unrestricted, Tygon, and PVC salamanders for whole body and specific body regions

		Body	Head	Trunk	Tail
25%	Model	**7.77E-08**	**1.69E-04**	**1.82E-04**	**3.61E-12**
	Tygon vs. PVC	**3.23E-08**	**7.94E-04**	1.68E-01	**5.85E-12**
	Unrestricted vs. PVC	1.26E-01	8.34E-01	**2.92E-03**	**4.01E-04**
	Unrestricted vs. Tygon	**5.08E-03**	**1.17E-03**	**1.36E-04**	4.85E-02
50%	Model	**1.52E-03**	**2.12E-09**	**1.55E-05**	**1.33E-03**
	Tygon vs. PVC	**2.68E-02**	**6.92E-04**	7.92E-01	**1.27E-03**
	Unrestricted vs. PVC	8.12E-02	**1.12E-03**	**1.52E-05**	7.83E-01
	Unrestricted vs. Tygon	**1.39E-03**	**1.03E-09**	**2.20E-04**	**2.66E-02**
75%	Model	**5.46E-06**	**7.52E-05**	**1.32E-02**	**3.86E-07**
	Tygon vs. PVC	**7.00E-06**	**6.70E-04**	9.11E-01	**1.90E-07**
	Unrestricted vs. PVC	9.95E-01	4.88E-01	**1.53E-02**	2.95E-01
	Unrestricted vs. Tygon	**6.64E-04**	**3.73E-04**	**2.63E-02**	**3.23E-03**

A general linear mixed model with individual as a random effect was performed and curvature values were assessed at 25%, 50%, and 75% of the stride cycle (α = 0.05). *P*-values are reported for the model, as well as pairwise post-hoc comparisons. Bold indicates significance.

Curvature in the head region is likely associated with head movements but these head movements do not appear to be related to significant changes in path direction (i.e., turning). There were significant differences between TYS individuals compared with PVCS and UR individuals at 25% and 75% of the stride cycle; TYS individuals had significantly higher absolute mean head curvature than both PVCS and UR individuals (Fig. 2D and F). Only at 50% of the stride cycle were there significant differences among all three treatments; TYS individuals had the highest absolute mean head curvature followed by UR and PVCS individuals (Fig. 2E).

The PVCS effectively restricted curvature in the trunk region. PVCS individuals had significantly lower absolute mean trunk curvature than both TYS and UR individuals at 25%, 50%, and 75% of the stride cycle (Fig. 2G–I). There were no significant differences in absolute mean trunk curvature between TYS and UR individuals at 25%, 50%, and 75% of the stride cycle. These results indicate that the TYS was flexible enough to permit typical lateral movements in the trunk region similar to UR.

Tail curvature increased in response to anything fastened to the trunk. PVCS and TYS individuals had significantly higher absolute mean tail curvature than UR individuals at 25%, 50%, and 75% of the stride cycle (Fig. 2J–L). Although absolute mean trunk curvature for TYS individuals was not significantly different than that of UR individuals, salamanders still increased lateral movements in their tails with these shells on.

### Kinematic variables

**Fig. 3:**
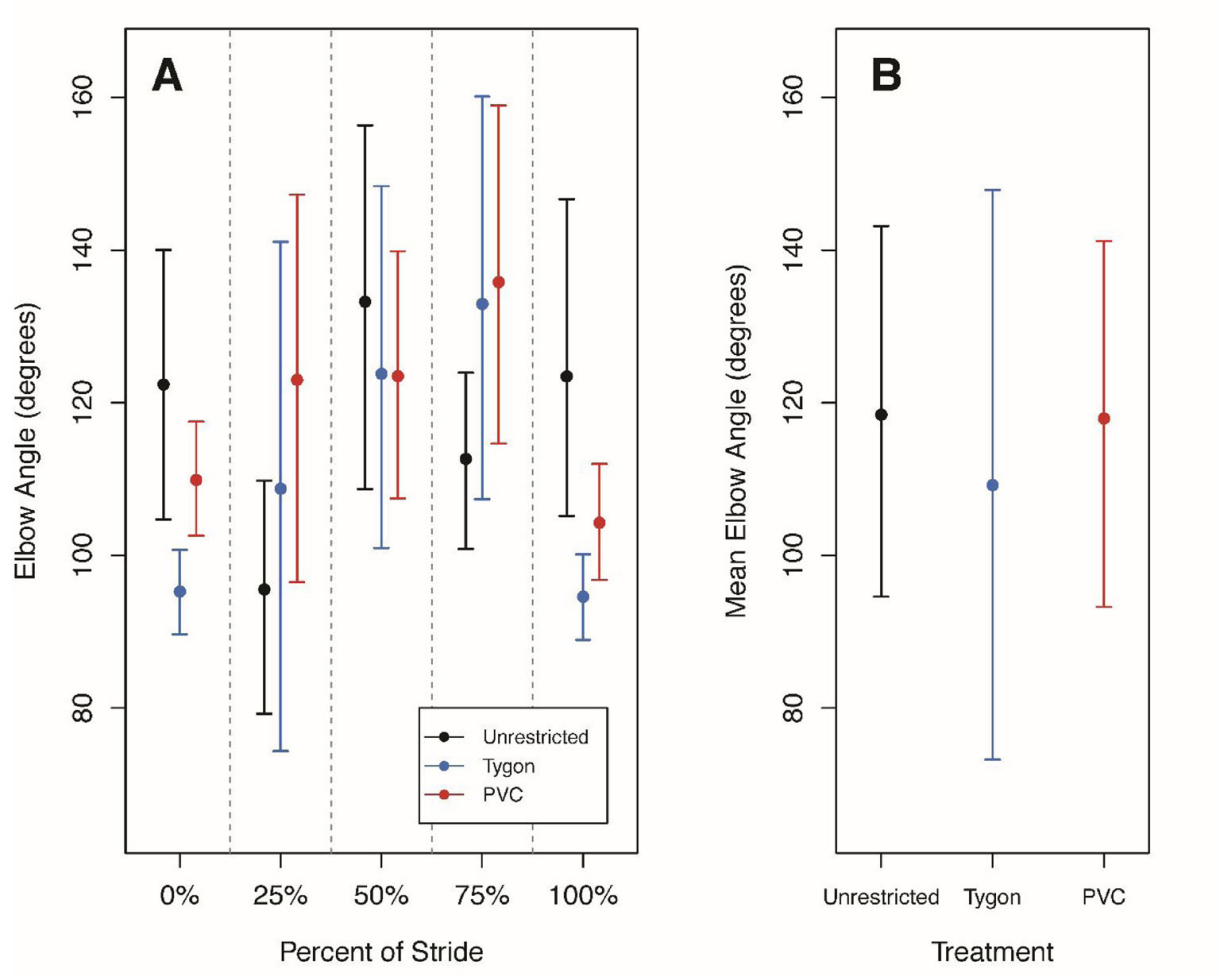
The mean elbow angle for Unrestricted, Tygon, and PVC salamanders at 0%, 25%, 50%, 75%, and 100% of the stride cycle and the overall mean elbow angle across the entire stride cycle. Plotted data are circular means with highest posterior density intervals as error bars. **(A)** The stride cycle was broken up into estimates at 0%, 25%, 50%, 75%, and 100% to determine whether there were differences among treatments. There were no significant differences in elbow angle among treatments at 25%, 50%, and 75% of the stride cycle. At 100%, TYS individuals had a significantly lower elbow angle than UR individuals. **(B)** Mean elbow angles across the entire stride cycle were also calculated for each treatment and there were no significant differences.

**Fig. 4:**
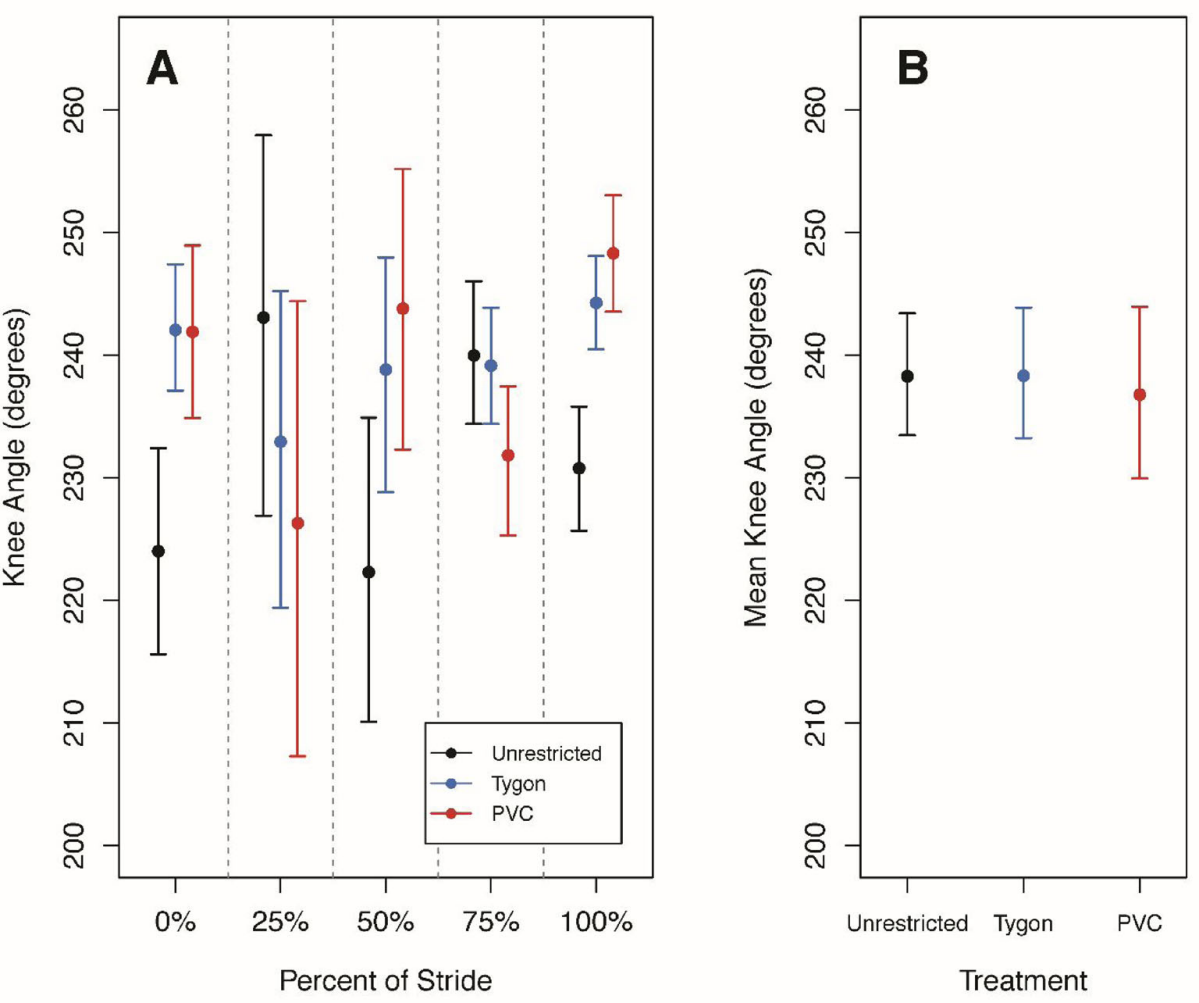
The mean knee angle for Unrestricted, Tygon, and PVC salamanders at 0%, 25%, 50%, 75%, and 100% of the stride cycle and the overall mean knee angle across the entire stride cycle. Plotted data are circular means with highest posterior density intervals as error bars. **(A)** The stride cycle was broken up into estimates at 0%, 25%, 50%, 75%, and 100% to determine whether there were differences among treatments. There were no significant differences in knee angle among treatments at 25%, 50%, and 75% of the stride cycle. At 0% and 100%, TYS and PVCS individuals had a significantly more obtuse knee angle than UR individuals. **(B)** Mean knee angles across the entire stride cycle were also calculated for each treatment and there were no significant differences.

There were no significant differences in mean elbow or knee angles at 25%, 50%, or 75% of the stride cycle among treatments (Figs. 3A and 4A; Table 4). At 0% and 100% of the stride cycle, there were significant differences for both mean elbow and knee angles. At 0%, TYS individuals had a significantly lower mean elbow angle than both PVCS and UR individuals (Fig. 3A) and at 100%, TYS individuals had a significantly lower mean elbow angle than UR individuals (Fig. 3A). At 0% and 100%, TYS and PVCS individuals had significantly more obtuse mean knee angles than UR individuals (Fig. 4A).

**Table 4:** Summary statistics from circular mixed-effects model performed using a Bayesian approach on mean limb angles across the stride cycle by treatment

Treatment	Angle	Circular mean	LB HPD	UB HPD
Unrestricted	Elbow	118.4509	94.5734	143.1280
	Knee	238.3089	233.4905	243.4335
Tygon	Elbow	109.2145	73.2590	147.8571
	Knee	238.3479	233.2424	243.8904
PVC	Elbow	117.9530	93.2511	141.1698
	Knee	236.8073	229.9572	243.9657

Posterior estimates of the circular means of the mean elbow and knee angles calculated across the stride cycle by treatment.

**Fig. 5:**
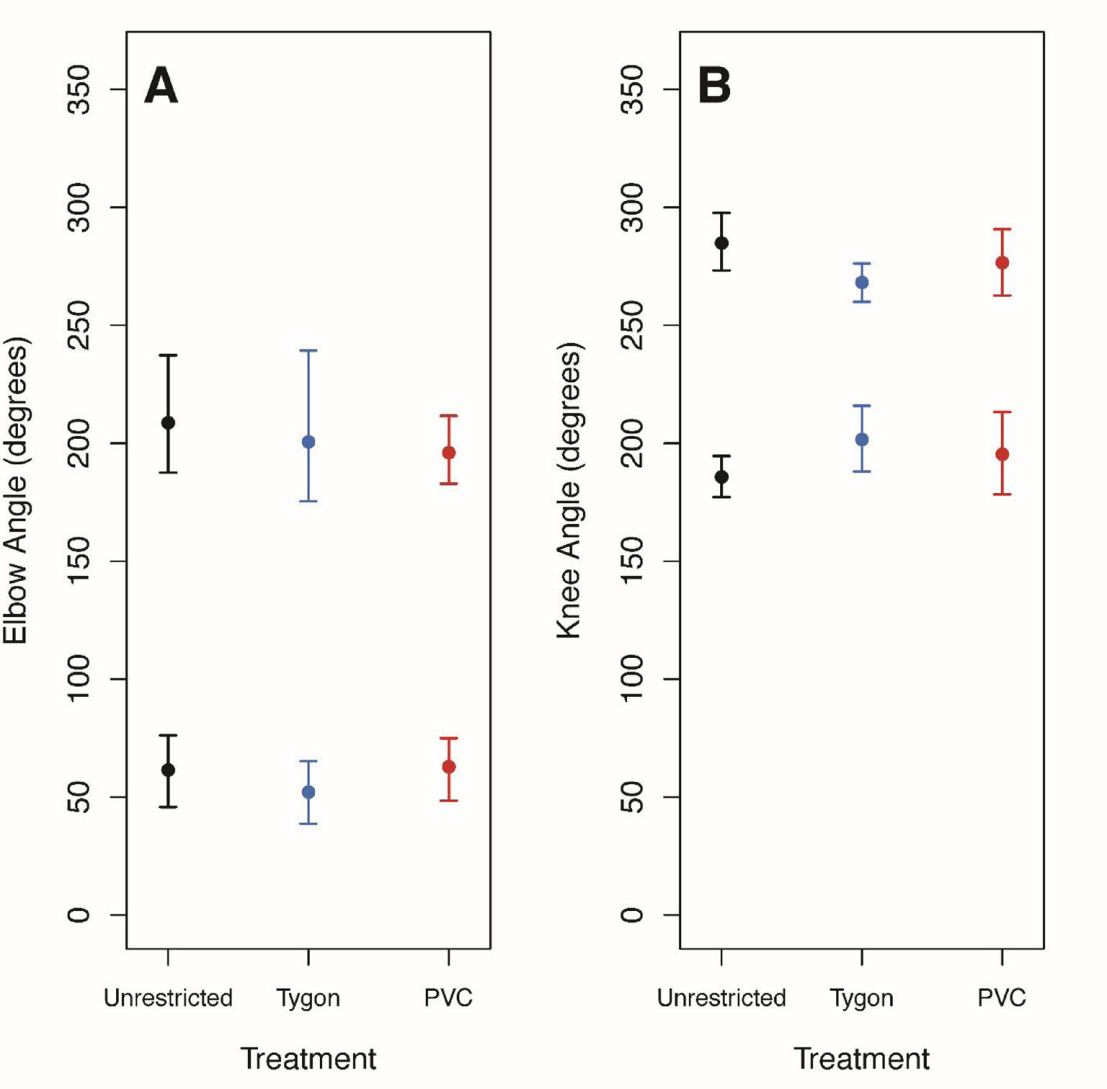
The mean minimum and maximum elbow **(A)** and knee **(B)** angles for Unrestricted, Tygon, and PVC treatments. Plotted data are circular means with highest posterior density intervals as error bars. (A) There were no significant differences among treatments for mean minimum or maximum elbow angles or (B) for mean minimum or maximum elbow angles.

The mean elbow and knee angles calculated for the entire stride cycle were not significantly different among treatments (Figs. 3B and 4B). Neither the mean minimum nor maximum angles for the elbow or knee were significantly different among treatments (Fig. 5A and B). There were also no significant differences in the range of angles for the forelimbs or hind limbs among treatments (Table 5).

**Table 5:** Summary statistics from circular mixed-effects model performed using a Bayesian approach

		Unrestricted			Tygon			PVC		
Variable	Angle	Mean	LB HPD	UB HPD	Mean	LB HPD	UB HPD	Mean	LB HPD	UB HPD
Estimate_0	Elbow	122.41	104.71	140.02	95.28	89.63	100.69	109.90	102.57	117.51
Estimate_25	Elbow	95.55	79.25	109.76	108.76	74.37	141.05	123.02	96.47	147.24
Estimate_50	Elbow	133.26	108.69	156.32	123.81	100.97	148.36	123.50	107.47	139.87
Estimate_75	Elbow	112.66	100.82	123.93	132.97	107.34	160.11	135.82	114.63	158.94
Estimate_100	Elbow	123.47	105.15	146.66	94.60	88.92	100.12	104.29	96.79	111.98
Y_min	Elbow	61.58	45.78	76.14	52.20	38.71	65.20	62.93	48.52	74.93
Y_max	Elbow	208.80	187.59	237.26	200.67	175.44	239.26	196.09	182.86	211.66
Range_Y	Elbow	136.03	97.75	176.79	132.88	92.10	179.72	128.76	97.28	162.21
Estimate_0	Knee	224.03	215.61	232.45	242.08	237.12	247.43	241.94	234.88	248.95
Estimate_25	Knee	243.10	226.92	257.95	232.96	219.41	245.26	226.32	207.27	244.42
Estimate_50	Knee	222.32	210.09	234.91	238.85	228.82	248.00	243.83	232.31	255.20
Estimate_75	Knee	240.01	234.41	246.04	239.18	234.41	243.90	231.85	225.30	237.47
Estimate_100	Knee	230.80	225.68	235.82	244.30	240.49	248.11	248.35	243.56	253.04
Y_min	Knee	185.80	177.20	194.63	201.68	188.07	215.94	195.43	178.31	213.23
Y_max	Knee	284.89	273.20	297.62	268.21	259.92	276.16	276.61	262.59	290.69
Range_Y	Knee	99.53	77.73	120.63	60.98	44.91	78.27	79.39	55.77	101.40

Posterior estimates of the circular means of the mean elbow and knee angles for all variables used in statistical analysis.

LB HPD: lower bound highest posterior density interval; UB HPD; upper bound highest posterior density interval.

## Discussion

Lateral movements of the vertebral column contribute significantly to locomotion in most sprawling tetrapods such as salamanders (Ijspeert 2020). In contrast, the bony carapace of turtles necessitated a reorganization of the tetrapod body plan that might have impeded locomotor performance. However, evidence suggests that despite their novel body plan, the locomotor capabilities of turtles are sufficient for their needs. Turtles successfully adapted for both terrestrial and aquatic environments and generalist species can navigate both habitats using kinematics for swimming and walking (Rivera and Blob 2010). Turtles also show diversity in their locomotor strategies for swimming with forelimbs both between modes of locomotion (i.e., rowing and flapping) and within a locomotor mode (Rivera et al. 2013; Blob et al. 2016). Turtle shells prevent lateral undulations because the vertebrae are fused to the carapace, so they depend solely on their limbs for propulsion. Turtles have been able to optimize their locomotor performance by increasing stride length via greater limb protraction (Schmidt et al. 2016), thus apparently compensating for the lack of lateral undulations. Differences among species in the dimensions and shape of the carapace and shoulder girdle affect how much shoulder girdle rotation contributes to stride length (Schmidt et al. 2016; Mayerl et al. 2019a). There is also evidence that this strategy of increasing limb protraction via girdle rotation to increase stride length applies to the hind limbs that are important for generating propulsion during both swimming and walking (Mayerl et al. 2016). Thus, despite the novelty of the turtle body plan, locomotor shoulder and pelvic girdle movements are still effective mechanisms for increasing stride length.

The PVC shell used in the present study effectively decreased curvature in the trunk region of salamanders. We hypothesized that salamanders would adjust their forelimb and hind limb kinematics in response to trunk lateral restriction. Specifically, we expected to see greater elbow and knee angles in PVCS individuals compared with UR and TYS individuals, similar to the limb movement seen in turtles in which axial movements are restricted by bony shells. Much of our kinematic data, however, indicate few such adjustments among the salamanders for our treatments. Salamanders responded by increasing the curvature in their tails, rather than through kinematic changes to limb movements, the strategy seen in turtles.

Although the trunk region of the salamanders in our study was restricted similarly to turtles, salamanders have proportionately longer tails that are actively used during locomotion. Nearly all turtle species have highly reduced tails that have typically been thought to play a limited role during locomotion. However, there are five extant species with long tails in Platysternidae and Chelydridae in which the tail might contribute to locomotor performance. During underwater walking, snapping turtles (*Chelydra serpentina*) generate a standing wave and move their tails like a strut much like salamanders do (Ashley-Ross and Bechtel 2004; Willey and Blob 2004). Willey and Blob (2004) argue that this pattern of tail movement in snapping turtles could be the posterior portion of a standing wave typical in terrestrial walking; in turtles, of course, the posterior portion of the wave is the only part that can be generated due to the presence of the shell. The role of snapping turtle tails during terrestrial walking and swimming has not been investigated, but this presents an interesting avenue for future studies to determine whether there are functional similarities between long-tailed turtles and salamanders. If this is true, the limb kinematics of snapping turtles should not show the same extent of protraction seen in other turtles that do not have much of a tail. However, there have been no limb kinematic studies on snapping turtles that we are aware of. In this study, salamanders may not have needed to adjust their limb kinematics because their tail undulations diminished the effects of the movement restriction in the trunk region.

Although the biomechanical role of tail movements is not well understood, especially in turtles, this mechanism has been investigated in leopard geckos (*Eublepharis macularius*) that lift their tails off the ground and swing them laterally during locomotion. Jagnandan and Higham (2017) compared leopard geckos with their original tail, a restricted tail with a rod attached, and an autotomized tail. While salamanders’ terrestrial movements are characterized by standing waves, leopard geckos initially generate a standing wave that changes to a traveling wave as it moves more posteriorly (Hamley 1990). It was previously thought that tail autotomy in lizards caused changes in locomotion because of a change in mass or shift in center of mass, but it appears that these changes are due to the loss of tail undulations during locomotion (Jagnandan et al. 2014). The loss of tail undulations caused the leopard geckos to adopt a more sprawling posture and decreased femur retraction and step length (Jagnandan and Higham 2017). It may not be surprising that there were no significant changes to salamander limb kinematics in our study, given that their tails were still mobile. Other studies also have shown that tail undulations during steady locomotion assist in responding to unexpected perturbations, stability, force generation by the caudofemoralis, and propulsive forces generated by the hind limbs (Reilly 1994; Irschick and Jayne 1999; Jagnandan and Higham 2017; Wang et al. 2018). Tail movements could serve as another mechanism of control that helps sprawling tetrapods overcome obstacles in challenging environments. Salamanders have both trunk and tail neural circuits capable of working both independently and in parallel, suggesting the axial motor system has some flexibility to respond to challenging environments (Charrier and Cabelguen 2013).

Our study determined that a reduction in lateral flexibility in the trunk did not result in limb kinematic changes during walking in salamanders. The salamanders in our study selected a range of speeds for a walking gait, and these speeds for both experimental treatments were lower than the range for the unrestricted treatment. A potential explanation is that the additional mass from the Tygon and PVC shells contributed to this limitation in their performance. In future studies, one could temporarily attach individual weights to the salamanders to determine whether, and at what additional mass, the speed of the salamanders is affected. This information about the effects of mass on salamander locomotor performance could be used to make new, lighter versions of shells that do not affect the performance of the salamander.

Another potential direction for subsequent studies is the role of restricted tail movements. Tail undulations can play a critical role in the locomotion of sprawling tetrapods, but we did not restrict or quantify tail movements in our study. It is possible the movement of the tail gave salamanders the ability to respond to the reduced lateral flexibility in their trunks, but further work is needed to quantify and investigate the mechanisms by which these movements contribute to locomotor performance. Other studies on tail undulations suggest that it may be the loss of movements in the tail that necessitates changes in limb kinematics.

## Data Availability

The data that support the findings of this study are available from the corresponding author upon request.
